# Primary nasopharyngeal Kaposi’s sarcoma without cutaneous manifestations: a case report

**DOI:** 10.1186/s13256-026-06190-w

**Published:** 2026-07-08

**Authors:** Emmanuel L. Lugina, Zakayo Magembe Ernesti, Emanuel Livin Nundu, Angela Tabian Mlole, Felister Tupa, Herriethsiah StanleyNoah, Chacha Josiah Mwita, Innocent J. Mosha, Salama Iddy, Crispin Andrew Kahesa, Owen Ngalamika, Charles Wood, Julius David Mwaiselage, Salum J. Lidenge

**Affiliations:** 1 Ocean Road Cancer Institute, P.O. Box 3592, Dar Es Salaam, Tanzania; 2 Muhimbili University of Health and Allied Sciences, Dar Es Salaam, Tanzania; 3 Muhimbili National Hospital, Dar Es Salaam, Tanzania; 4 Dermatology and Venereology Division, University Teaching Hospital, University of Zambia School of Medicine, Lusaka, Zambia; 5 Department of Interdisciplinary Oncology, Louisiana State University Health Sciences Center, New Orleans, LA USA

**Keywords:** Kaposi’s sarcoma, Nasopharynx, Acquired immunodeficiency syndrome, Sub-Saharan Africa

## Abstract

**Background:**

Kaposi’s sarcoma (KS) is a neoplastic lymphovascular disorder. It typically appears on the skin of the upper and lower extremities, but can also occur on the mucosal surfaces of the head and neck. The most common site reported for head and neck injuries is within the oral cavity, particularly on the palate, and other mucosal sites, such as the nasopharyngeal area. Moreover, involvement of the nasopharynx without skin involvement is even rarer.

**Case presentation:**

Here, we present a rare case of HIV associated/epidemic KS in a 30-year-old man of African origin, where the primary manifestation was in the nasopharyngeal (NP) area. After the diagnosis, the patient began chemotherapy with the ABV regimen and low-dose radiotherapy, which resulted in clinical improvement and no recurrence during the 6-year follow-up period.

**Conclusion:**

We have reported the first case of primary nasopharyngeal epidemic Kaposi’s sarcoma (KS) in SSA, which presented at an unusual location with atypical features on routine hematoxylin and eosin (H&E) examination and without cutaneous manifestations. The unusually high median survival for this poor-risk epidemic nasopharyngeal KS points to the need to study the natural history of non-cutaneous KS in comparison to other forms of KS that have demonstrated a very dismal survival. This case is highly representative of the complexity of HIV management. The authors aim to bring awareness of the KS's unusual locations and diagnosis challenges.

## Introduction

Kaposi’s sarcoma (KS) is a low-grade vascular tumor associated with infection with human herpesvirus 8 (HHV-8), also known as the KS-associated herpesvirus (KSHV) [1]. Therefore, KS prevalence reflects the prevalence of KSHV infection [2]. KSHV seroprevalence varies considerably worldwide. It ranges from 69 to 90% in Uganda [3], 10–30% in Mediterranean countries (for example, Sicily or Sardinia), and less than 6% in the USA and Switzerland [4]. The exact routes of KSHV transmission are currently unclear, although the highest levels of KSHV shedding are found in saliva [5]. Studies from KSHV-endemic African countries have revealed a high KSHV seroprevalence in infants, indicating horizontal transmission of KSHV between parents and children and between siblings in these regions [6]. Thus, saliva could serve as a medium for KSHV transmission, compatible with both sexual and non-sexual pathways [4].

The disease is named after Moritz Kaposi, an Austro-Hungarian dermatologist on the faculty of the University of Vienna, who first described the entity in 1872 as “idiopathic multiple pigmented sarcoma of the skin” [7]. The most affected sites are the skin and the mucous membrane [8]; however, it can be found in other parts of the body, like the lungs, stomach, and intestines [9–11]. The head and neck region involvement is more prevalent in the Human Immunodeficiency Virus (HIV)/AIDS-associated form (epidemic KS), with the oral cavity being the most commonly affected mucosal site [12, 13]. Head and neck KS may appear as a primary mucosal or cutaneous tumor [14]. Cutaneous involvement in the head and neck region is present in about 66% of cases, mucosal forms are seen in 56%, and enlarged lymph nodes are present in 13% [15]. Other head and neck KS sites apart from the oral cavity include the skin, oropharynx, tongue, hypopharynx, and larynx. The conjunctiva, lacrimal gland, parotid gland, masseter muscle, or tonsils are affected in rare instances [16]. Primary involvement of the nasal cavity, particularly the nasopharynx, is extremely rare, with only a few reports in the literature [13, 14, 16–24].

Visceral KS without cutaneous manifestation is rare in both the pre-HAART and the HAART era [25]. Here, we present a case of an epidemic nasopharyngeal KS without cutaneous lesions treated with HAART, radiotherapy, and chemotherapy. Indeed, to our knowledge, this is the first reported case from sub-Saharan Africa (SSA).

## Case presentation

A 30-year-old male of African origin with no significant medical or family history of chronic illnesses presented with a six-month history of progressive nasal obstruction and difficulty in swallowing. Further questioning revealed nasal regurgitation and a change of voice. He denied any history of fever or night sweats. There was no history of hemoptysis, hematemesis, nasal bleeding, or melena. He denied smoking, alcohol abuse, and weight loss.

During the physical examination, the patient was alert and oriented to person, place, and time. He had neither cutaneous nor oral lesions. He had no palpable lymphadenopathy or splenomegaly. He had a hot potato voice and a Karnofsky Performance Status (KPS) score of 80. His vitals showed a normal blood pressure of 120/72 mmHg, sinus tachycardia at around 140–150 beats per minute, and a temperature of 36.5 °C.

**Fig. 1:**
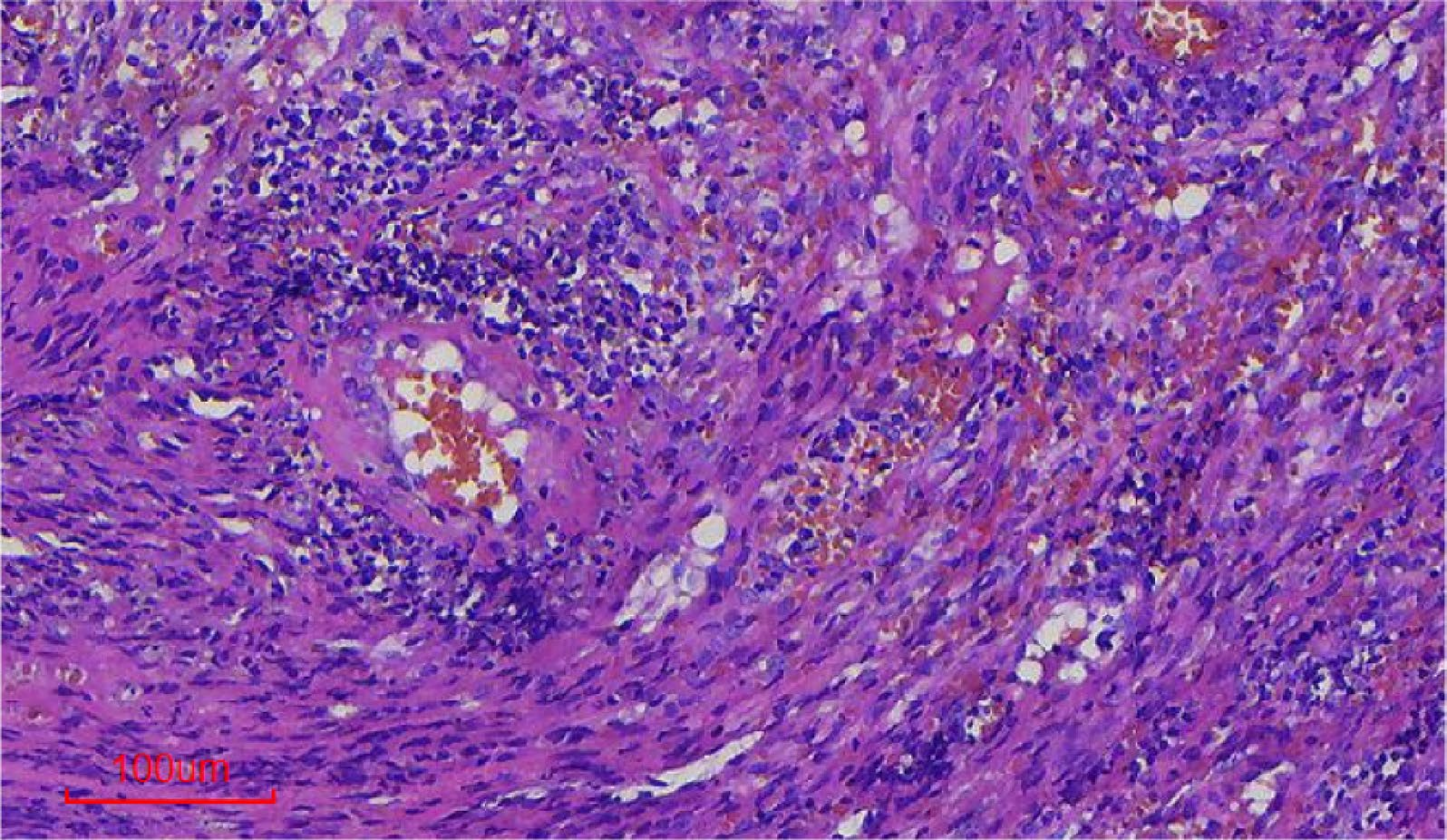
Hematoxylin and eosin (H&E) stain

With a clinical differential diagnosis of nasopharyngeal carcinoma, we referred him to Ear, Nose, and Throat (ENT) surgeons. Nasopharyngoscopy showed a bulky lesion extending from the nasopharyngeal (NP) area. No contact bleeding was seen, and a biopsy of the mass was performed. Hematoxylin–eosin stain was performed, and the examination revealed a lot of inflammatory lymphoid infiltrates, pieces of upper respiratory tract-type mucosa with a cellular tumor in the subepithelial stroma, with spindle cells and vascular slits displaying mild nuclear atypia (Fig. 1)*.*

**Fig. 2:**
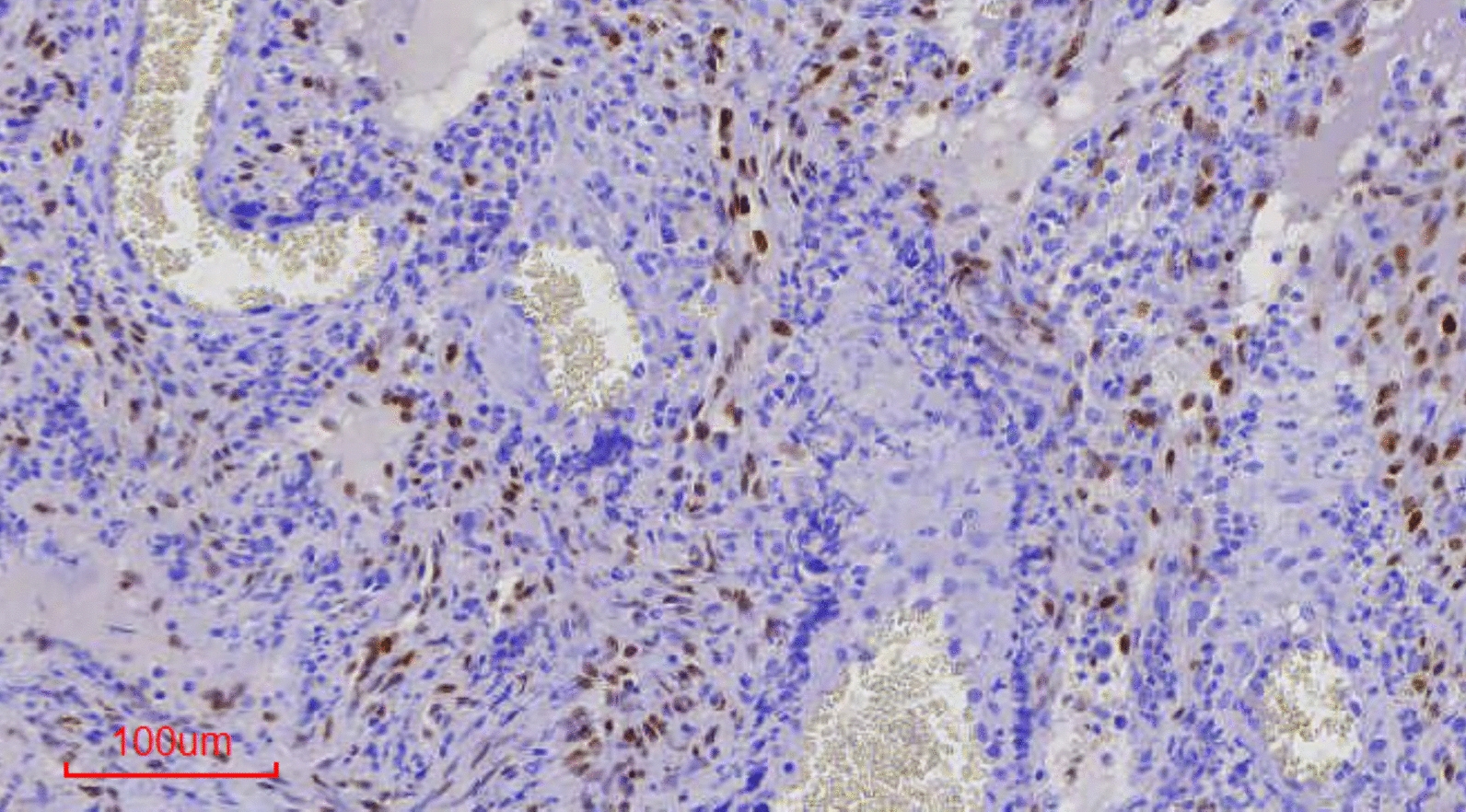
Latency-associated nuclear antigen (LANA) immunohistochemistry, original magnification × 40

Kaposi’s sarcoma-associated herpesvirus (KSHV) latency-associated nuclear antigen (LANA) immunohistochemistry was performed to confirm the diagnosis of KS. The characteristic spindle cells showed punctate nuclear staining for KSHV (Fig. 2)*.* Immunohistochemical staining for the endothelial cell markers CD31 and CD34 was not performed.

Upon diagnosis, the patient underwent further investigations and was found to be HIV-1 serology positive with a CD4 count of 76 cells/µL. Other laboratory investigations revealed a hemoglobin level of 12 gm/dL and a total white blood cell count of 6000 cells/mm^3^. Liver and renal function tests were normal. Serology for HBsAg and VDRL were negative. Unfortunately, the HIV viral load was not documented at the time of diagnosis. The diagnosis of KS in the presence of HIV infection advanced his disease to acquired immunodeficiency syndrome (AIDS). A final diagnosis of the epidemic nasopharyngeal KS ACTG stage T111S0 (poor risk) was rendered.

**Fig. 3:**
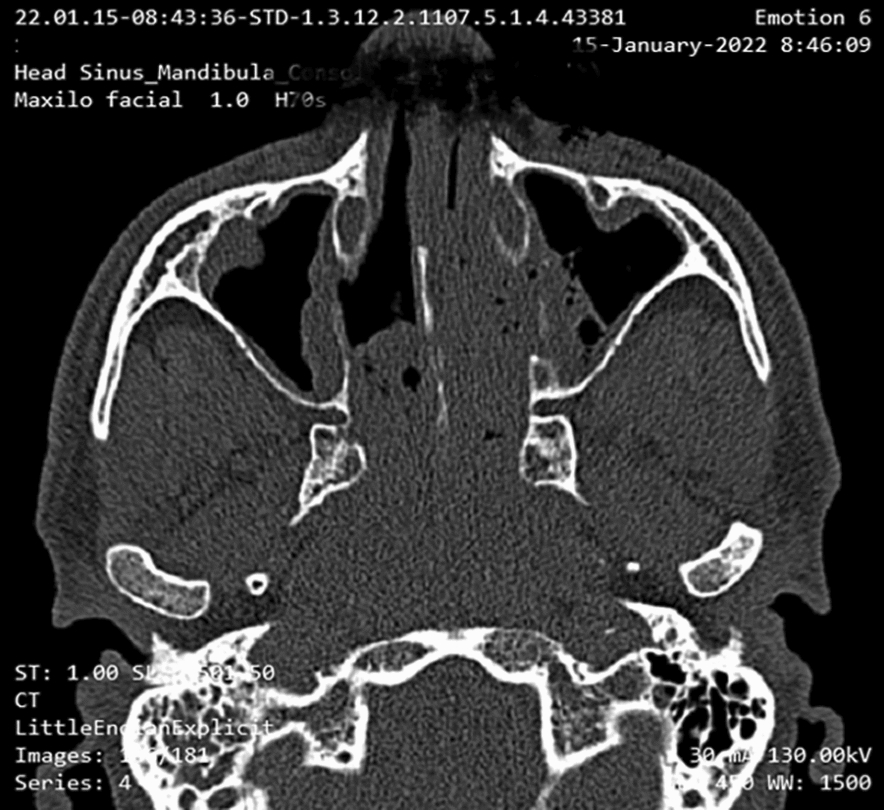
Computed tomography scan of the head: before treatment

Staging computed tomographic scans demonstrated an extensive NP mass measuring (8.1 × 5.4 × 7.2) cm, likely from the right fossa of Rosen Muller crossing the midline and insinuating bilateral nasal cavity more on the left and extending to the oropharynx and pushing the soft palate downward. No significant abnormality was detected in the thorax, abdomen, and pelvis (Fig. 3)*.*

The multidisciplinary consultation meeting decided to put the patient on highly active antiretroviral therapy (HAART) followed by chemotherapy because surgical excision was not feasible. The patient began a TLD regimen (tenofovir disoproxil, lamivudine, and dolutegravir) as per the National HIV treatment guideline [26] under the HIV care and treatment (CTC) monitoring.

**Fig. 4:**
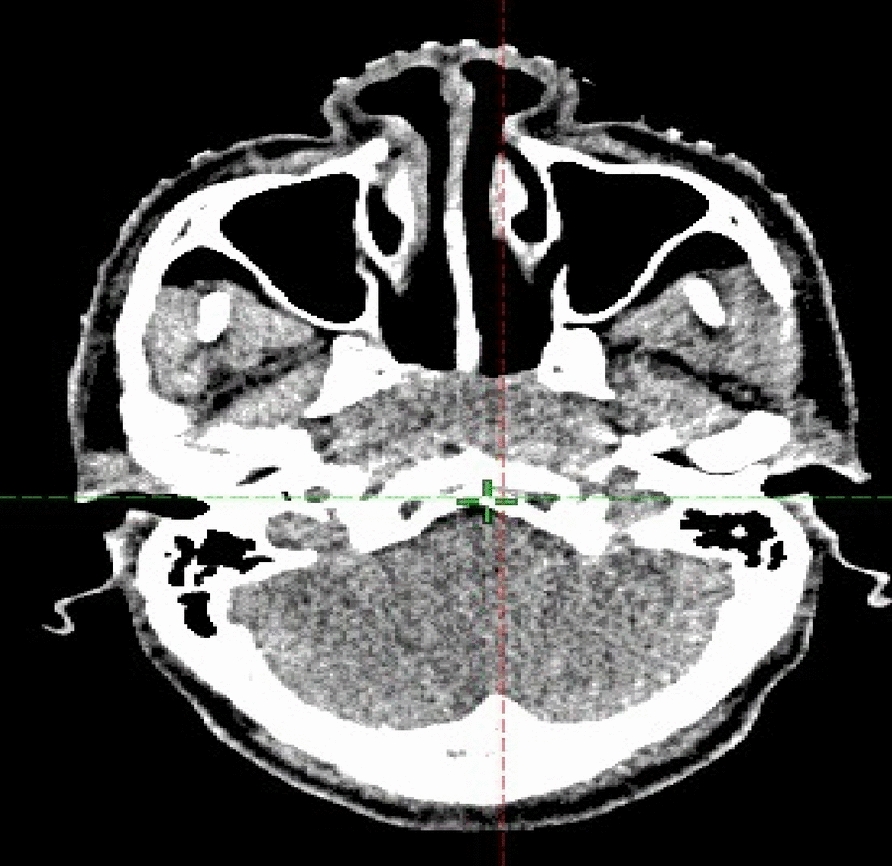
Computed tomography scan after three cycles of chemotherapy

**Fig. 5:**
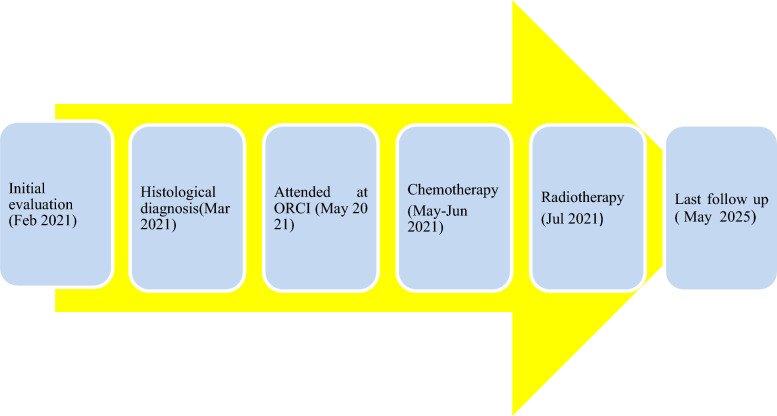
Timeline of significant events

Because of impending upper airway obstruction, high-risk KS ACTG stage, and the lesion's visceral nature, he was treated with three cycles of chemotherapy (ABV regimen) from May to August 2022 as induction chemotherapy. He tolerated chemotherapy well without major side effects. He was in partial response after the three cycles, as evidenced by an interim CT scan of the head (Fig. 4)*.* Chemotherapy was followed by consolidation with 6 MV photons 3D conformal radiotherapy (22 Gy in 11 fractions) in August 2022, which led to the resolution of all symptoms. He developed grade 3 mucositis during radiotherapy, which was managed by magic mouthwash (BMX) and resolved within two weeks after finishing radiotherapy. He could not afford another CT scan after finishing radiotherapy to assess the treatment response; however, he has remained well three years after the initial diagnosis with no symptoms or signs of recurrence. ENT evaluation during follow-up visits has remained unremarkable. The care of this patient is summarized in Fig. 5, which includes both historical and current information.

## Discussion

We report on a rare case of nasopharyngeal primary KS without skin involvement in a newly diagnosed HIV-positive individual. We believe this is the first reported case in sub-Saharan Africa.

Our patient presented with nasal regurgitation. Nasal regurgitation is observed in more than 35% of patients with head and neck tumors [28]. Nasal regurgitation is a common symptom among individuals with oropharyngeal dysphagia [29]. Our patient also presented with a hot potato (thick and muffled) voice. A 'hot potato' voice may indicate severe upper airway obstruction caused by a space-occupying lesion in the pharynx and larynx [30].

Although KS lesions have a distinctive appearance, histologic diagnosis is challenging due to their variable morphologic features, which depend on the stage of the disease and range from patchy to nodular, and the presence of various histological variants [31]. The diagnosis becomes even more challenging when KS occurs in an unusual location, such as the nasopharynx. During the patchy phases of the disease, the irregularly shaped, slit-like vascular spaces and spindle cell proliferation may seem unnoticeable. The heavy reactive lymphoid infiltration in the nasopharyngeal region, as observed in this case report, may obscure it, which may be particularly prominent in HIV patients [27]. It may be mistaken for reactive or benign vascular proliferations, such as pyogenic granuloma or bacillary angiomatosis, which can result from an infection with *Bartonella henselae* or *Bartonella quintana* [24]. Over 50% of benign non-epithelial nasopharyngeal lesions are caused by vascular tumors, with juvenile angiofibroma being the most prevalent and having spindle cell proliferation [28]. In the advanced phases, whereby the spindle cell proliferation is prominent, a spectrum of vascular-forming spindle cell neoplasms of the nasopharynx needs to be considered, such as a solitary fibrous tumor, nasopharyngeal angiofibroma, sinonasal glomangiopericytoma, and Kaposiform hemangioendothelioma [27]. It is necessary to include differential diagnoses of KS, such as EBV-associated smooth muscle tumor [35] and mycobacterial spindle cell pseudotumor [36], in the clinical evaluation of individuals living with HIV and spindle cell proliferation. While most KS have low-grade nuclear features, anaplastic KS can have marked nuclear atypia or pleomorphism [30], which could result in incorrect diagnosis of aggressive tumors like angiosarcoma or nasopharyngeal squamous carcinoma. Anaplastic KS can also be confused with spindle cell carcinoma, a rare variant of squamous cell carcinoma [30].

KS's key diagnostic features include spindle cells, slit-like vessels, extravasated red blood cells, and hyaline globules. As we have shown, the diagnosis of KS can only be confirmed when vascular markers such as CD31, CD34, and ETS-related gene (ERG) are co-expressed with KSHV. It is essential to exclude KSHV expression from the aforementioned differential diagnoses [29]. The presence of KSHV can be confirmed with immunohistochemistry for LANA immunohistochemistry [28].

No generally accepted KS staging exists, even for individual subtypes [29]. In addition to staging, according to Mitsuyasu and Groopman [30] and the AIDS Clinical Trials Group (ACTG) TIS staging system [31] for epidemic HIV-associated KS, the European KS guidelines categorize KS into management-relevant situations. The various KS subtypes require individualized investigations, staging, and management based on symptoms, course, and extent of involvement. According to the European KS guideline, three situations are distinguished for managing KS patients: local non-aggressive KS, local aggressive KS, and disseminated KS [36]. The ACTG TIS staging system can be used to classify patients into good- and poor-risk categories based on tumor extent (T), the severity of immunosuppression (I), and the presence of any other systemic HIV-associated illness (S) [32]. Histopathologically, KS progresses through three continuous stages: patch, plaque, and nodule. Each stage features distinctive gross and microscopic features [33]. Our patient was diagnosed with nodular high-risk KS, representing an advanced stage of the disease.

The fundamental basis for treating HIV-associated KS is to suppress HIV replication by starting antiretroviral treatment and treating opportunistic infections. HAART has the potential to significantly reduce the incidence of KS, slow its progression, and even cause pre-existing diseases to regress [39]. There is no clear evidence that certain ART regimens are superior to others (40), but some good-risk KS patients are likely to exhibit tumor regression with HAART alone [32]. In our case, HAART was initiated following HIV diagnosis; however, due to the advanced nature of the lesion and the associated impending airway obstruction, it was decided that chemotherapy was needed to shrink the tumor and prevent airway obstruction.

Local therapeutic options are primarily indicated for local disease and include excision surgery, cryotherapy, radiotherapy, alitretinoin gel, intralesional chemotherapy (typically vinblastine, but vincristine or interferon-alpha can also be used), laser therapy, and photodynamic treatment [32]. Radiotherapy is one of the local treatment options for all KS subtypes [36] because most KS tumors are radiosensitive. Radiotherapy is preferred for treating localized tumors and has been successfully used in various areas, including the skin, oral cavity, lungs, and conjunctiva. An objective response rate of 74% was observed in a retrospective study of HIV-associated KS patients treated with radiotherapy [17]. Relatively low radiotherapy doses are necessary for effective treatment, allowing radiation to be delivered to acral areas of the face with minimal side effects and good cosmetic results. Fractioning options include 1 × 8 Gy, 5 × 4 Gy, or 12–20 × 2 Gy, depending on the indication, patient performance status, size, and lesion extent. Generally, small lesions can be treated with hypofractionated doses, while more extensive lesions should be irradiated more frequently with lower fractionation doses [36]. There is controversy in the optimum fractionation of radiotherapy; however, regardless of fractionation, the complete response rate (CR) is ~62.5%, and the partial response rate (PR) rate is ~30%, resulting in an overall response rate (OR) of ~92.5% [34]. Our patient was treated with low-dose conventional fractionation radiotherapy because of the location of the lesion to minimize toxicity to the surrounding critical organs.

Chemotherapy can potentially treat multiple diseases, but it can also cause severe systemic side effects [17]. In resource-rich settings, pegylated liposomal doxorubicin and paclitaxel are highly effective in inducing regression of advanced HIV-associated Kaposi sarcoma. Although paclitaxel appears more effective than liposomal anthracyclines, its high toxicity makes it a second-line therapy (41). In resource-limited countries such as Tanzania, where liposomal doxorubicin or daunorubicin is currently unavailable, and paclitaxel is unaffordable, a more toxic combination chemotherapy regimen with adriamycin, bleomycin, and vincristine (ABV) is widely used, as in this case report. The CR and PR with paclitaxel are ~ 15.9% and ~ 63.7%, respectively, giving an OR of 79.6% [35]. The OR with the ABV regimen is 88% [36], while with pegylated liposomal doxorubicin, it is 76% [37].

The optimal number of chemotherapy cycles for effective therapy is three to six. However, the actual length of chemotherapy treatment depends upon the KS's response to therapy. To treat all microscopic KS, chemotherapy may be administered for 1–2 cycles after achieving complete remission. If the KS lesions shrink but do not disappear, chemotherapy can continue as long as it is tolerated and the KS does not grow [37]. Systemic chemotherapy should be reserved for patients in whom local therapy fails or who have extensive disease [38].

Refractory cases can be treated with immunomodulatory drugs. Objective response rates have been observed in approximately 30% of patients administered subcutaneous interferon-alfa (43). Imatinib showed an OR of 33% (44). Thalidomide resulted in a 40% OR with a median duration of response of 7 months (45). The overall OS for bevacizumab was 31% [39], while pomalidomide had an OR of 73% (46). Pembrolizumab showed an OS of 60.7% (47). Most of these studies were small phase 2 clinical trials.

Visceral KS can occur anywhere in the viscera, particularly in the lymph nodes, lungs, and gastrointestinal tract. While visceral lesions are found in 33–77% of patients with cutaneous KS at autopsy, they are rare when there are no cutaneous manifestations [31]. Our patients had visceral KS without cutaneous lesions. The absence of skin lesions in some patients with visceral KS in the HAART era is still puzzling, and several questions remain as to whether this form of KS is distinct from that with cutaneous manifestations. Little is known about the host–tumor interaction in such patients and what limits cutaneous manifestations in a newly diagnosed and HIV-untreated patient. Importantly, the characterization of outcomes in such patients is warranted, as the median survival for most cutaneous KS in Africa is approximately six months. In contrast, our case has shown KS remission for almost six years since diagnosis. This contrasts with a study by *Stebbing *et al*.*, which found that visceral KS without cutaneous manifestations had noninferior treatment outcomes compared with cutaneous KS in the HAART era [25].

The lack of pre-treatment and post-treatment HIV viral loads limits our case report, and a post-treatment CT scan could not be done.

## Conclusion

We have reported the first case of primary nasopharyngeal epidemic Kaposi's sarcoma (KS) in SSA, which presented at an unusual location with atypical features on routine H&E examination. This highlights the importance of performing a comprehensive clinical and diagnostic workup to confirm the diagnosis and avoid any unnecessary delay in initiating appropriate therapy. The unusually long survival of this poor-risk epidemic nasopharyngeal KS suggests the need to study the natural history of non-cutaneous KS compared to other forms of KS, which have demonstrated very dismal survival rates.

## Data Availability

Data are available upon reasonable request.
